# Synthesis of microspheres of triuranium octaoxide by simultaneous water and nitrate extraction from ascorbate-uranyl sols

**DOI:** 10.1007/s10967-013-2763-9

**Published:** 2013-10-08

**Authors:** M. Brykala, A. Deptula, M. Rogowski, W. Lada, T. Olczak, D. Wawszczak, T. Smolinski, P. Wojtowicz, G. Modolo

**Affiliations:** 1 Institute of Nuclear Chemistry and Technology (INCT), 16 Dorodna Str., 03-195 Warsaw, Poland; 2 Forschungszentrum Jülich GmbH, Institut für Energie-und Klimaforschung, Nukleare Entsorgung und Reaktorsicherheit (IEK-6), Jülich, Germany

**Keywords:** Uranium oxides, Sol–gel, Nuclear fuel, Spherical particles

## Abstract

A new method for synthesis of uranium oxide microspheres (diameter <100 μm) has been developed. It is a variant of our patented Complex Sol–Gel Process, which has been used to synthesize high-quality powders of a wide variety of complex oxides. Starting uranyl-nitrate-ascorbate sols were prepared by addition of ascorbic acid to uranyl nitrate hexahydrate solution and alkalizing by aqueous ammonium hydroxide and then emulsified in 2-ethylhexanol-1 containing 1v/o SPAN-80. Drops of emulsion were firstly gelled by extraction of water by the solvent. Destruction of the microspheres during thermal treatment, owing to highly reactive components in the gels, requires modification of the gelation step by Double Extraction Process—simultaneously extraction of water and nitrates using Primene JMT, which completely eliminates these problem. Final step was calcination in air of obtained microspheres of gels to triuranium octaoxide.

## Introduction

Triuranium octaoxide is the principal precursor of nuclear fuel in the form of natural or enriched in isotope of ^235^U. In actually generation of nuclear reactors, finally uranium dioxide powder is synthesized by various routes (e.g. thermal decomposition and reduction U(VI)–U(IV) of simple uranium substrates, such as uranium trioxides, ammonium poliuranates or uranyl nitrate) via triuranium octaoxide. If enriched UF_6_ is used, then the defluorinating step is necessary followed by further processing to UO_2_. Finally UO_2_ powders are pressed to pellets and after sintering loaded into fuel rods.

The spherical shape of nuclear fuels is proposed for IV generations of nuclear reactors (e.g. high temperature gas cooled reactors) [1]. Unique method to fabrication them is sol–gel process. It is the wet chemical method which mainly involves the gelation of a droplet of sol to the desired fuel material into a gel microsphere. Afterwards, they are washed, dried and heat treated to obtain high density microsphere [2, 3].

Complex Sol–Gel Process (CSGP) elaborated in INCT [4] has been used for synthesis variety of advanced ceramic materials e.g. HTC [4], hydroxyapatite [5], Li_2_TiO_3_ [6] etc. Recently we used it for synthesis of uranium dioxides [7, 8]. Starting sols have been prepared by addition of ascorbic acid to uranyl nitrate solution followed by controlled alkalization. Sols were gelled to irregularly shaped powders by evaporation of water, or to medium sized spherical particles with diameter below 100 μm by applying so called IChTJ Process (INCT in English) [9]. It is worth to mention that first solid complexes of uranyl ion with ascorbic acid has been synthetized by Veselinović and Śusić [10], but without any intention to uranium dioxide production.

However using described CSGP when we started from uranyl nitrate solution we observed difficulties in gelation process by IChTJ process and farther thermal treatment. The goal of this work was modification of process for elimination this problems.

The goal of the work is elaborating of method of synthesis of precursor to oxide nuclear fuels—as triuranium octaoxide, which in the final step of production will be reduced to uranium dioxide. Application of CSGP allows of obtaining this precursors in the spherical shape (with diameter below 100 μm), what is necessary for nuclear fuels for advanced nuclear reactors.

## Experimental

The following reagents were used: uranyl nitrate (Chemapol Praha, >99 %), ascorbic acid pharmaceutical grade (Takeda Europe GmBH), 2-ethylhexanol-1 (Acros Organics, 99 %), SPAN-80 (Fluka), and Primene JMT-commercial name of aliphatic amines, principally C_18_H_37_N_2_ (Fluka). All other reagents used were of p.a. grade.

Gels and final products were characterized by the following methods: Thermogravimetric analysis (TG) and differential thermal analysis (DTA) with a Hungarian MOM Derivatograph, scanning electron microscope (SEM) observation with a Zeiss DSM 942 and Jeol JSM64-90LV.

**Fig. 1:**
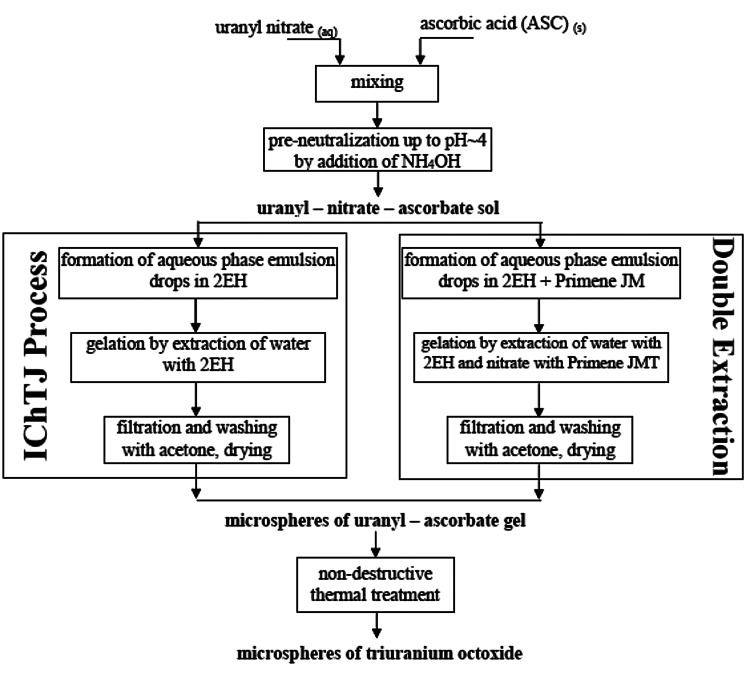
Flowchart of preparation of microspheres of triuranium octaoxide by CSGP with different gelation steps

The flowchart of applied combination of CSGP and IChTJ processes to synthesis of spherical particles of uranium dioxide is shown in Fig. 1.

The first step of the method is formation of complex—uranyl-ascorbate sol solution (molar ratio ASC/U = 1) by addition of a very strong complexing agent as ascorbic acid (ASC)—it is the salient feature of CSGP process (according to the Eq. 1). Afterwards, partial hydrolysis by addition of ammonia-solution to a certain pH value before precipitation (pH ~4), is carried out.

1 $$ {\text{UO}}_{2}^{2 + } + HA \to {\text{UO}}_{2} {\text{A}}^{ + } + {\text{H}}^{ + } $$

A^−^ is the deprotonated ascorbic acid.

**Fig. 2:**
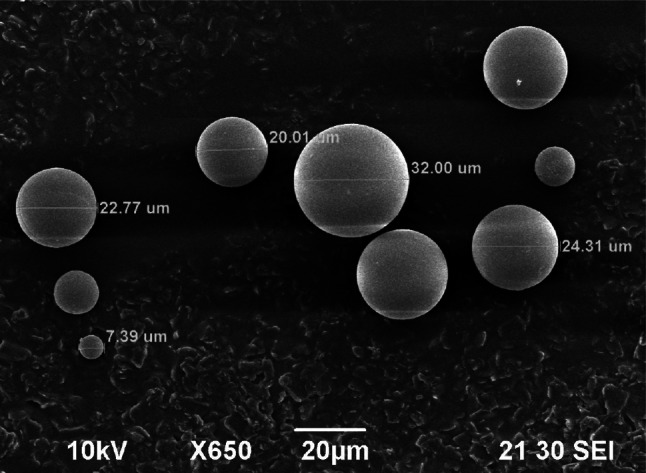
SEM of spherical particles of ascorbate-uranyl gel (MR ASC/U = 1)

Next step, which is crucial to obtain the desired shape of final product, is gelation of complex sol. Those sols can be gelled into irregularly agglomerates by addition of higher amount of ammonium hydroxide (gelation agent) at whole volume, and to spherical particles (Ø above 200 μm) by internal gelation [11] or medium sized spherical particles (Ø below 100 μm) by modified External Gelation—IChTJ process (Fig. 1). The resulting ascorbic-hydroxy-uranyl sol is added very slowly upon very vigorous stirring to an organic solvent selected from a group which consists of long-chain aliphatic alcohols, such as *n*-octanol, preferably 2-ethylhexanol-1 (EH) which contains 1 vol.% of surfactant—sorbitol monooleate (SPAN-80). The gelling process is terminated when after switching off the stirrer, the brown–violet spherical grains which are falling to the bottom have no tendency to stick to one another and to create larger aggregates. Subsequently, the spherical gel powders are filtered, washed with acetone. The products, after this part of method, are shown on Fig. 2.

The ICHTJ method as external gelation process is designed to fabricate of spherical particles only to diameter 100 μm. The reason is non-fully extraction of water from larger drops of sol. Then gelation is only on the surface of drops and in the inside of drops is still non gelled sol solution. The final step of a combination of CSGP and IChTJ processes is non-destructive thermal treatment (with spherical shape retention)—calcination to U_3_O_8_.

## Description of the problem

**Fig. 3:**
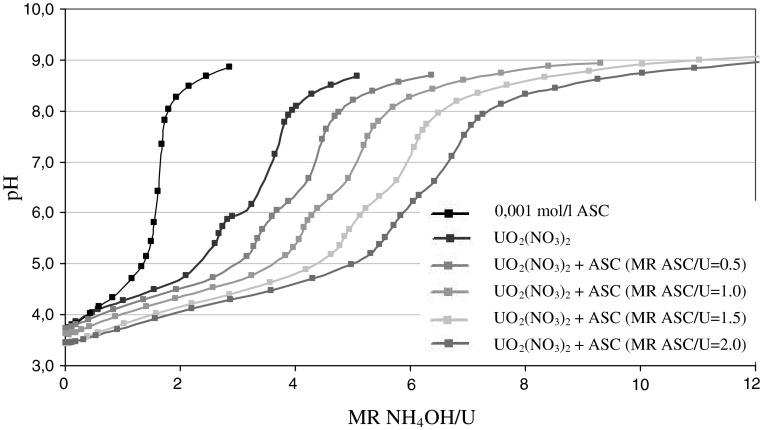
Potentiometric titration of 0.001 M UO_2_(NO_3_)_2_ with ammonia (0.1 M). *Note* *violet line* show titration of titration of 0.001 mol/l ASC with ammonia. (Color figure online)

The gelation to spherical particles of complex—uranyl-nitrate-ascorbate sol solution needs partial hydrolysis by addition of ammonia-solution to a certain pH value before precipitation (pH ~4). The omission of addition of ammonia prevents of obtaining spherical particles of gel. Curves of potentiometric titration of various ascorbate-uranyl sols (0.001 M) obtained from uranyl nitrate, with ammonium hydroxide (0.1 M) are shown in Fig. 3. Given the role played by the gelation agent in the method CSGP aqueous ammonia solution, it is impossible to get the full curves of the initial solutions pH changes in a wide range, because in pH above value 4 gelation of sol solution to solid gel is observed. Therefore, the measurements were carried out for the diluted solutions. In the sample without ascorbic acid, we can observe a small plateau in the pH region ~6 (MR NH_4_OH/U = 3) connected with polymerization of uranyl ion to polinuclear ions followed by definite increase of pH (inflection point approx. MR = 3.5) representing formation of ammonium poliuranates in the solution. Those effects are considerably masked with an increase of ASC concentration, which confirms strong complexing ability of this reagent. Evidently, a value of inflection points is proportionally higher with increase of MR ASC/U.

**Fig. 4:**
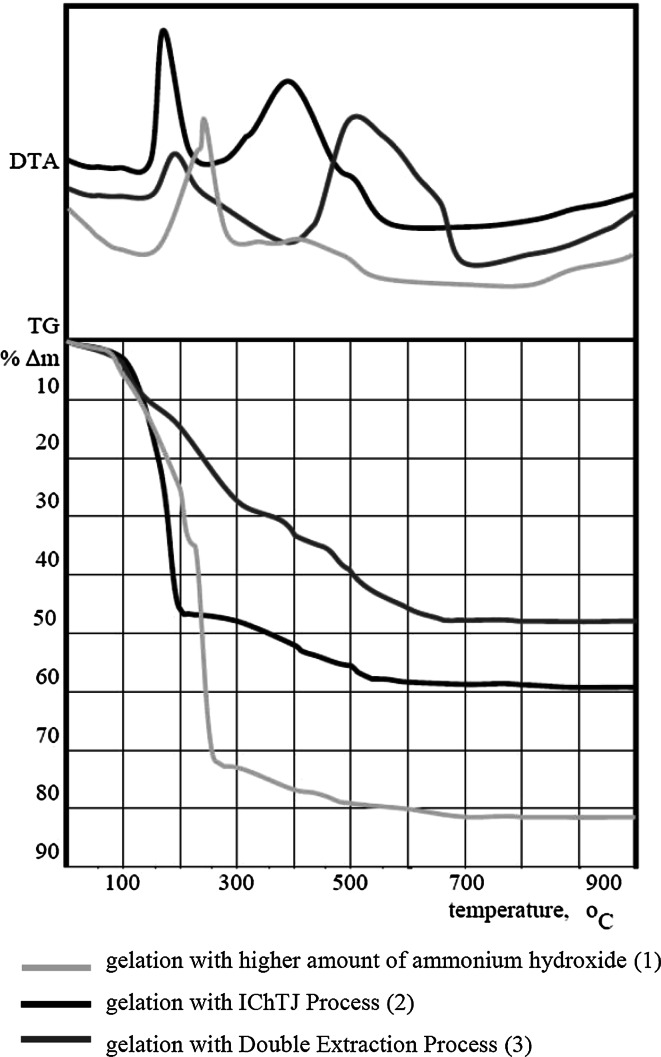
Thermal analysis (TG, DTA) of ascorbate-uranyl gels

First step of preparation of sol solutions has very big influence for the next steps of fabrications of spherical particles of uranium oxides. In particular influences for thermal treatment to triuranium octaoxide, which is developed on the basis of thermal analysis, shown in Fig. 4.

**Fig. 5:**
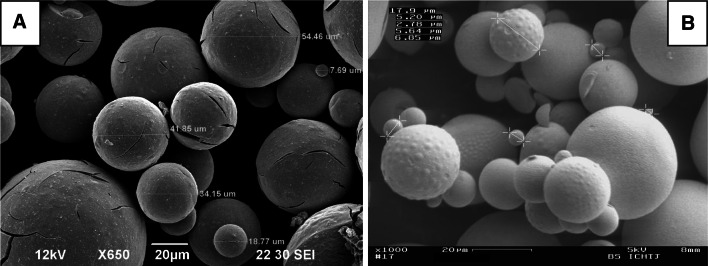
SEM of triuranium octaoxide in shape of *spherical* particles obtained by: **a** IChTJ Process, **b** Double Extractions Process

First weight loss of all obtained gels in temperatures 100–300 °C is connected to evaporation of molecular water and hydroxide groups, but also it is also connected to decomposition of the U-ASC-NO_3_–NH_4_OH gel, when one of component—ammonium nitrate—decomposes violently (see curve no. 1) [12]. The result is obtained of chipped spherical particles (Fig. 5a). The next weight loss with broad exothermic effect 350–550 °C, is connected with combustion of ASC and products of its decomposition. Only on curve no. 1 there is not observed exothermic effect of decomposition of organic compound. It is due to producing of high amount of energy in first exothermic effect what causes earlier decomposition of ascorbic acid.

The essence of the problem is obtaining of crushed particles or cracked surface of particles, caused by violently decomposes of ammonium nitrate in higher temperature (170–250 °C). Ammonium nitrate is obtained during the time of pre-neutralizing by ammonium hydroxide of ascorbate-uranyl sol solution, when the uranyl nitrate is used as a source of uranium.

## Results

Thermal analysis of gels (Fig. 4, lines nos. 1 and 2) produced from uranyl nitrate shows that thermal treatment is complex process and it requests special procedures to avoid “explosions” in calcination step. First possibilities is modify procedure of thermal treatment for slower and longer heating. In the first step, the heating rate was low (1 °C/min) with stop in 250 °C per 1 h. Next step of thermal treatment is the elimination of organic compounds at ~700 °C.

Second option is using new method of gelation by Double Extraction Process—simultaneously extraction water with nitrate (Fig. 1) and finally obtained of microsphere of gels with lower content of nitrates. This process has been used in INCT for synthesis spherical powders of strontium zirconate and described in [13].

Starting sols, prepared as in Fig. 1, were emulsified and gelled in 2EH containing 2v/o Primene JMT. It was observed that gelation time has been several times shorter than for 2EH without Primene JMT. Thermal analysis shown in Fig. 4 (curve no. 3) shows lower exothermic effect at ~250 °C then for samples contained original nitrate content (curve no. 1). On the basis of weight loss we estimated that about 10 % of nitrates were extracted relative to IChTJ Process. In contrast the second exothermic effect (400–700) is stronger. It means that quantity of ASC and its decomposition products is higher than in gels containing more nitrates. After that weight is stable and represents the formation of triuranium octaoxide (U_3_O_8_), see Fig. 7.

**Fig. 6:**
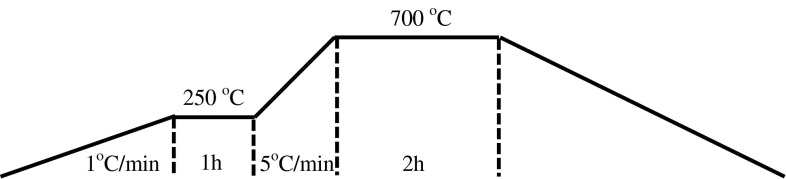
Flow charts of calcination of uranyl-ascorbate-hydroxyl gels to triuranium octaoxide

We observed that the microspheres remained intact during heating, even when using relatively high (10 °C/min) heating rates (Fig. 5b). These positive results are the effect of lower concentration of nitrates in U-ASC-NO_3_–NH_4_OH, and consequently much less content of potentially “explosive” ammonium nitrate (Fig. 6).

**Fig. 7:**
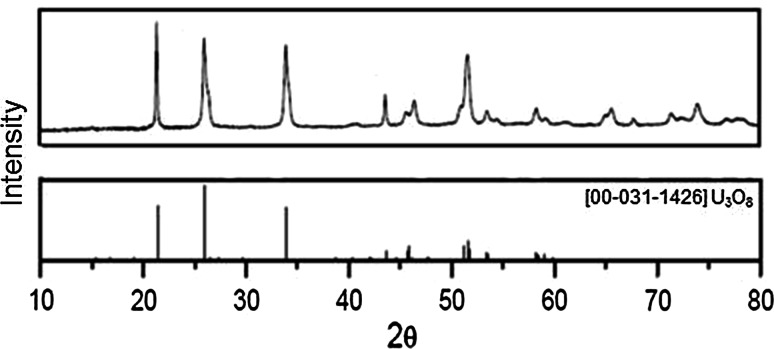
XRD analysis of uranyl-ascorbate gels after calcinations (700 °C/2 h)

Broad exothermic effect 400–700 °C, is connected with combustion of ASC and products of its decomposition. After that weight is stable and represents the formation of triuranium octaoxide (U_3_O_8_), see Fig. 7.

X-ray diffraction analyses were done after the thermal treatment of gels prepared from uranyl nitrate. The results of XRD analysis (Fig. 7) show that after this step there is observed the expected mainly triuranium octaoxide forms.

## Conclusion

A new technique of gelation of uranyl sols obtained from uranyl nitrate has been elaborated. It focus on simultaneously extraction water and nitrate anions, what in the results allows produce a microspheres of gels with lower content of nitrates. This modification (Double Extraction Process) eliminates the serious disadvantage as destruction of the microspheres during thermal treatment which is connected with violent decomposition of ammonium nitrates, formed in time of alkalization of uranyl-nitrate-ascorbate sol by ammonium hydroxide. Then, even relatively high heating rates (10 °C/min) can be used.

It is necessary to underline that preparation of uranyl gels from uranyl nitrate by extraction of nitrates without addition of ascorbic acid is not possible.
